# Role of Estradiol Hormone in Human Life and Electrochemical Aptasensing of 17β-Estradiol: A Review

**DOI:** 10.3390/bios12121117

**Published:** 2022-12-02

**Authors:** P. P. Waifalkar, Daegwon Noh, Poorva Derashri, Sagar Barage, Eunsoon Oh

**Affiliations:** 1 Department of Physics, Chungnam National University, Daejeon 34134, Republic of Korea; 2 Institute of Quantum Systems, Chungnam National University, Daejeon 34134, Republic of Korea; 3 Amity Institute of Biotechnology, Amity University, Mumbai-Pune Expressway, Panvel 410206, Maharashtra, India; 4 Centre for Computational Biology and Translational Research, Amity University, Mumbai-Pune Expressway, Panvel 410206, Maharashtra, India

**Keywords:** 17β-estradiol, electrochemical aptasensor, nanomaterials, aptamer immobilization, blocking layer

## Abstract

Estradiol is known as one of the most potent estrogenic endocrine-disrupting chemicals (EDCs) that may cause various health implications on human growth, metabolism regulation, the reproduction system, and possibly cancers. The detection of these EDCs in our surroundings, such as in foods and beverages, is important to prevent such harmful effects on humans. Aptamers are a promising class of bio-receptors for estradiol detection due to their chemical stability and high affinity. With the development of aptamer technology, electrochemical aptasensing became an important tool for estradiol detection. This review provides detailed information on various technological interventions in electrochemical estradiol detection in solutions and categorized the aptasensing mechanisms, aptamer immobilization strategies, and electrode materials. Moreover, we also discussed the role of estradiol in human physiology and signaling mechanisms. The level of estradiol in circulation is associated with normal and diseased conditions. The aptamer-based electrochemical sensing techniques are powerful and sensitive for estradiol detection.

## 1. Introduction

**Figure 1:**
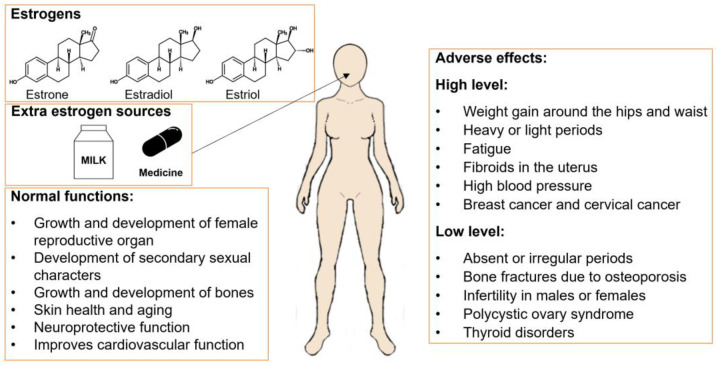
Types of estrogen and role of estradiol.

Estradiol is a form of the most recognized and powerful steroid hormone known as estrogen. Estradiol is often called the “female hormone,” as it regulates the development and maintenance of female sex characteristics. Estradiol is one of the structurally comparable trio of estrogens (Figure 1). While estrone has only one -OH group and estriol (E3) has three -OH groups, estradiol has two -OH groups [1]. Estradiol is known as the most potent estrogenic endocrine-disrupting chemicals (EDCs) that may cause serious health implications on growth (premature puberty), metabolism, and reproduction, and possibly cause cancers (ovarian or breast) [2]. Furthermore, a variety of chemical compounds, such as synthetic hormones, agrochemicals, and industrial compounds, recognized as estrogenic act as effective ligands to induce hormonal response. Among them, synthetic hormones (Diethylstilbestrol (DES), 17α-ethinylestradiol, (17αEE2), and phytoestrogens), agrochemicals (atrazine, dieldrin, and toxaphene), surfactants (alkylphenol ethoxylates (APEOs)), and industrial compounds, including the phthalates, styrenes, polybrominated biphenyls (PBB), bisphenol A (BPA), dioxin (2,3,7,8-TCDD), nonylphenols (NP), octylphenols (OP), and polychlorinated biphenyls (PCB), act as EDCs. [3].

The detection of these EDCs in environments, e.g., foods and beverages, is essential to protect human beings. Many techniques used for 17β-estradiol (E2) sensing include high-performance liquid chromatography [4], gas chromatography/mass spectroscopy [5], and surface-enhanced Raman spectroscopy [6,7]. However, these devices have several limitations, such as difficulty in miniaturization, complicated operation, matrix effect, and high purchase and operation costs.

The electrochemical detection method has several advantages, such as low detection limit, reproducibility, and fast detection speed [8]. In electrochemical sensing, the redox reactions occur when reactant molecules and electrodes are in close proximity. Therefore, the adsorption of analyte molecules to the electrode, along with diffusion of the analyte molecules, plays an important role in the reaction rate. Electro-catalytic materials and artificial receptors, such as molecular imprinted polymers, etc., can be utilized to enhance the redox reaction and to improve the selectivity. Bio-receptors also provide improvements in reaction rate and selectivity. In this case, the analytes bind to the bio-receptors, and the bindings occur faster because the binding energies of the bio-receptors are generally greater than the adsorption energy.

Bioreceptors for electrochemical estradiol sensors, such as human estrogen receptor-based sensors, enzymatic sensors, and immunosensors, are well-known [9]. Even though enzymes have a high degree of selectivity for their substrates, they have several drawbacks, such as the large molecule size, the active site buried deep inside the structure, and the short shelf life, which reduce the efficiency of electron transfer [10].

One of the efforts to overcome these difficulties is using an aptamer as a bio-receptor. An aptamer is a kind of affinity ligand, composed of oligonucleotides. Aptamers act as “antibodies”, which have attracted the most attention among affinity ligands. An aptamer shows a high affinity toward its target [11]. Therefore, aptasensors are in demand in the biomedical field due to their extraordinary properties of low limit of detection, specificity, and reproducibility [12]. They can be developed within a short period for the desired target in vitro. Unlike general antibodies, they are produced in vitro through a synthetic device and have high performance. They also have the advantages of high stability, long-term preservation, low variation between batches, and high affinity for the target. Over the last decade, electrochemical aptasensors have attracted much attention, and a very impressive detection limit up to a femtomolar level has been reported for the detection of hormones, including E2 [13].

For E2 electrochemical aptasensing, choosing proper aptamers for E2 and aptamer binding to electrodes are necessary. With proper functional group modification (e.g., -SH) of the aptamers, we can utilize the chemical bonds (e.g., Au–S) for aptamer loading to electrodes. Depending on the aptamer binding strategy, the electrode material can be decided, or vice versa.

In this review, we investigate various available research results in electrochemical E2 detection. We start this review with the physiological and pathophysiological role of estradiol in Section 2. In Section 3, we introduce the basic principles of electrochemical sensing, and in Section 4 we compare the electrochemical detection performances without using aptamers. Then, in Section 5, which is the main part of this review, we group the available references by aptasensing mechanisms, electrode materials, and aptamer immobilization.

## 2. Estradiol in Human Physiology and Pathophysiology

Estrogens are steroid hormones that govern the human reproductive system’s growth, development, and function. Estrogens play a role in the neuroendocrine, skeletal, adipogenesis, and cardiovascular systems. Among the estrogens, estrone is the most prevalent hormone during menopause and is the most prevalent hormone during pregnancy. During the reproductive years, estradiol is the predominant estrogen.

**Figure 2:**
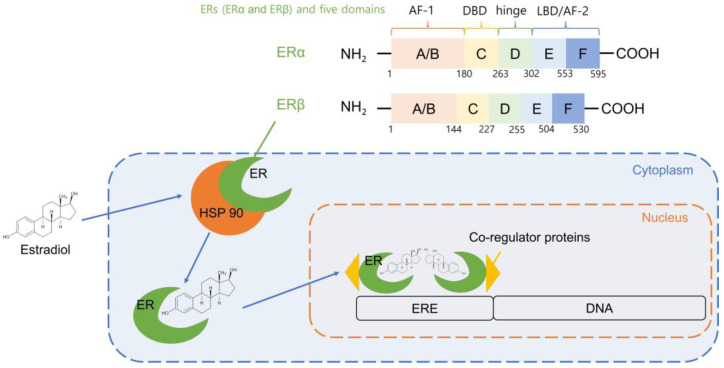
Estrogen receptors (ERs) and estrogen response elements (EREs).

Estrogen signaling pathways are selectively promoted or inhibited based on the balance of estrogen receptors (ERs) or ER activity in target organs. ERs belong to the steroid hormone superfamily of nuclear receptors, as transcription factors (Figure 2). After binding to estrogen, ERs influence biological activities, such as reproductive organ development, bone modeling, cardiovascular system functioning, metabolism, and behavior in both females and males through regulating gene expression [14].

When estrogen levels were ablated by ovariectomy and then treated with exogenous estrogen, researchers could understand the overall physiological roles of ERs. In addition, the involvement of estrogen signaling in general physiologic processes has been discovered by utilizing knockout mouse models of estrogen receptor alpha (ERα) and estrogen receptor beta (ERβ) functions. It has been observed that the biological roles of ERα and ERβ functions are unique in knockout mouse models. Thus, knockout mouse models are valuable tools for researching the roles of these genes under certain disease situations, even though they do not always give consistent results.

The extensive research on the structural and functional role of ER revealed that estrogen’s biological effects are mediated by its binding to the estrogen receptors (ERs), estrogen receptor alpha (ERα) and estrogen receptor beta (ERβ). Estrogen signaling is selectively activated or inhibited in target organs based on a balance of ERα and ERβ activity. In 1986, ER was cloned from MCF-7 human breast cancer cells, and in 1996, ER was cloned from rat prostate cells [15].

ERα and ERβ are both encoded by separate genes on different chromosomes. The ERα gene in humans is found on chromosome 6, while the ERβ gene in mice is found on chromosome 14. The full-length human ERα protein contains 595 amino acids and a molecular weight of 66 kDa, whereas the shorter version, ERβ, has 530 amino acids and a molecular weight of 54 kDa (ERβ). ERs, similar to other nuclear receptors (NRs), have five discrete domains with different roles (Figure 2). Activation function 1 (AF1), which contributes to ER transcriptional activity and is a critical domain for interaction with co-regulators, is found at the N-terminus of the A/B domains of ERs [16].

With only a 30% similarity between ERα and ERβ, AF1 is the least conserved area. ERβ has modest levels of AF1 activation, according to functional investigations. The A/B domains also include amino acids that are candidates for post-transcriptional changes, such as splicing, to enhance AF1 activity. The C domain encodes a DNA binding domain that is required for the sequence-specific binding of ERs to DNA and the regulation of target gene expression.

The D domain, which functions as a hinge region, contains amino acid sequences that increase nuclear localization signaling and facilitate ER post-translational modification, resulting in ER signaling activation in cells. Finally, the E/F domain comprises a ligand-binding domain in the C-terminal region that acts as a contact site with co-regulators as well as ligand-dependent activation function 2 (AF2). Since ER is activated by cellular reactions to the environment, AF1 and AF2 govern the transcriptional regulatory activity of ERs.

The E/F domains of ERα and ERβ have a 53 percent sequence similarity and influence cellular responses via ligand-dependent ER activation. ERα and ERβ activity is likewise influenced by the F domain [17]. The ability of ERs to selectively modulate the transcriptional activity of specific target genes may be influenced by changes in the F domain of ERs. Because of their interaction with heat shock protein 90 (Hsp90), ERs stay inactive in the absence of hormones. Protein stabilization, receptor binding affinity to ligands, and signaling cascades are all regulated by Hsp90 in eukaryotic cells. Hsp90 prevents inactivated ERs from the binding ligand and limits the degradation of unbound ERs. ERs are phosphorylated after binding to the ligand, form homo- or heterodimers, and subsequently, translocate into the nucleus. By attaching to estrogen response elements (EREs) in the DNA sequence (Figure 2), ERs control the transcription of target genes [18,19,20]. Because ERs bind to EREs, DNA bends and loops, allowing interaction with the transcriptional machinery and co-regulator proteins. Co-activators, co-repressors, co-integrators, histone acetyltransferases and deacetylases, and general transcriptional factors are all examples of co-regulators. Extracellular stimuli cause ER/co-regulator complexes to act specifically on target genes in certain organs. The distribution of ERs in different tissues shows that ERs have high specificity for the target tissue. The uterus, prostate stroma, ovarian theca cells, Leydig cells in the testes, epididymis, breast, and liver all have high levels of ER. The prostate epithelium, testes, ovarian granulosa cells, bone marrow, and brain all have high levels of ER. As previously stated, the downstream transcriptional activities of ERα and ERβ vary, resulting in tissue-specific biological responses [21,22,23]. The development and regulation of the female reproductive system are directly attributed to the steroid hormone estrogen (Figure 1). The neuroendocrine, skeletal, adipogenic, and cardiovascular systems are all affected by high levels of estrogen. Furthermore, estradiol is thought to be carcinogenic and to promote tumor growth. Its correlation with the risk of breast cancer is proven. The abnormal level of estradiol in women is associated with ovarian, endometrial, and lung cancers, even though the precise process is not yet fully understood. It is also widely acknowledged that estrogen levels in women can influence such conditions as weight gain, depression, exhaustion, mood changes, and difficulty in sleeping [24,25]. As a result, addressing the effects of estradiol levels in women may benefit from an estradiol test or from hormone therapy that is prescribed by a doctor.

It is well-known that the ecosystem is contaminated by estradiol distributed in the environment caused by the release of a significant amount of natural estradiol from human urine. Moreover, EDCs include synthetic hormones, agrochemicals and industrial compounds, which have been recognized as estrogenic and are frequently found in the environment. [3]. The effects of estrogenic compounds on the environment and food supply are well-known. It is commonly known that estradiol pollution kills and malforms fish, birds, and other wildlife, as well as humans [8]. The European Union’s Water Framework Directive (WFD) specifically identified E2 as an estradiol priority pollutant. Therefore, the identification and quantification of estradiol are crucial for scientific and health reasons related to biomedicine and environmental health. High-performance liquid chromatography and gas chromatography/mass spectroscopy methods are very sensitive and accurate in identifying estradiol, but these methods require expensive equipment and skilled personnel to measure and analyze estradiol.

## 3. Fundamentals of Electrochemical Sensing

**Figure 3:**
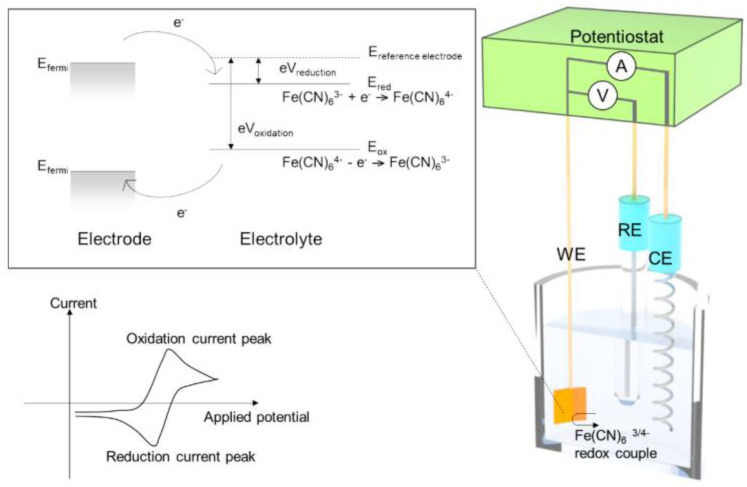
A schematic diagram for electrochemical redox reaction of Fe(CN)_6_^3−/4−^ in a three-electrode system.

Electrochemistry deals with the energy conversion between chemical energy and electrical energy. Electrochemical reactions can be controlled by an electrical potential applied on the electrode and be measured by the current through the electrode (Figure 3). To ensure accurate control and measurement of the potential, the three-electrode system with working (WEs), reference (REs), and counter electrodes (CEs) is commonly used. Figure 3 shows energy levels (electrochemical potentials) for the $\mathrm{Fe}{\left(\mathrm{CN}\right)}_{6}^{3-}+e^{-}↔\mathrm{Fe}{\left(\mathrm{CN}\right)}_{6}^{4-}$ redox reaction in a three-electrode system. If the fermi-level of a metal electrode is higher than the reduction energy level (E_red_) of Fe(CN)_6_^3−^, electrons move from the WEs to Fe(CN)_6_^3−^ and reduction occurs. On the other hand, if the fermi-level of a metal electrode is lower than the oxidation energy level (E_ox_) of Fe(CN)_6_^4−^, electrons move from Fe(CN)_6_^4−^ to WEs. As electrons transfer between the WEs and the redox species, ions in the solution move to compensate for the charge, forming a closed circuit of current [26]. The redox reaction can be measured as a current flow between the WEs and CEs.

Among various electrochemical techniques, cyclic voltammetry (CV) is a commonly used method for electrochemical detection. In the CV method, the working electrode potential changes triangularly as a function of time, and one can quickly measure the redox potentials during the CV measurements. Square wave voltammetry (SWV) and differential pulse voltammetry (DPV) are pulse voltammetry techniques, where the current difference at pulse edges is calculated. In the pulse voltammetry, the charging current can be removed and only the Faraday current can be measured. Electrochemical impedance spectroscopy (EIS) is a method to analyze the electrochemical reaction using an equivalent electric circuit. In the impedance analysis, an equivalent circuit model is commonly used, where the circuit components consist of charge transfer resistance, electric double-layer capacitance, and Warburg diffusion impedance.

## 4. Electrochemical Estradiol Sensing

The potential vs. current characteristic of an electrochemical reaction can be used for chemical sensing. However, it is difficult to detect trace-level estradiol with bare electrodes, such as Au electrodes and glassy carbon electrodes (GCE). Various modifications on working electrodes have been conducted to improve the sensitivity of electrochemical measurements. Using graphene, carbon nanotubes, polymers, and nanoparticles, E2 oxidations were successfully monitored thanks to their high conductivity, large surface area, catalytic properties, and electrode–estradiol interactions. Table 1 shows the electrode materials, detection limits, and the linear response range from several studies in which estradiol was detected by measuring its oxidation current.

On the other hand, probe materials, such as Fe(CN)_6_^3−/4−^ and methylene blue (MB), can be utilized for sensors using bio-receptors, such as antibodies and aptamers. In this case, the probe materials undergo redox reactions instead of the estradiol molecules. From the redox reaction, the presence and amount of estradiol can be estimated based on the fabricated sensor structures and mechanisms. EIS is one of the widely used techniques to analyze impedance changes as a function of the estradiol concentrations based on estradiol binding to bio-receptors. Voltage-current measurement techniques, such as CV and DPV, are also commonly used for the same purpose.

## 5. Electrochemical 17β-Estradiol Aptasensing

### 5.1. Aptamers for Estradiol Sensing

In most diagnostics and biomolecular sensing studies, high affinity and specific molecular recognition are achieved using antibodies, but there are some limitations, including animals or cell line requirements with complicated purification steps for antibody production. Unlike antibodies, selected aptamers can be reproducibly synthesized in vitro, which is convenient and also economical. Aptamers can bind to a wide range of substances, including proteins, peptides, amino acids, nucleotides, drugs, carbohydrates, and other small organic and inorganic molecules [33]. Antibodies may not be suitable for developing biosensors for environmentally toxic chemicals compared with aptamers. Moreover, aptamers can be modified with certain functional groups and are immobilized on many surfaces, both directly and indirectly, resulting in well-ordered receptor layers. Finally, aptamers are more stable than antibodies and, thus, are more resistant to denaturation and degradation.

In light of this, aptamers provide an advantageous alternative to antibodies as detecting molecules, particularly in the creation of a biosensor for chemicals [34]. Additionally, it was known that DNA aptamers are more stable than RNA aptamers and easier to identify and handle [35]. For E2 aptasensors, aptamers with various DNA sequences were tested by electrochemistry after attaching these aptamers to electrodes [36]. Nowadays, aptamers with various sequences are commercially available and researchers can choose the different functional group modifications with sequences such as -OH, amine, thiol, biotin, etc. Supplementary Table S1 summarizes the electrode materials and aptamer sequences reported to date.

**Figure 4:**
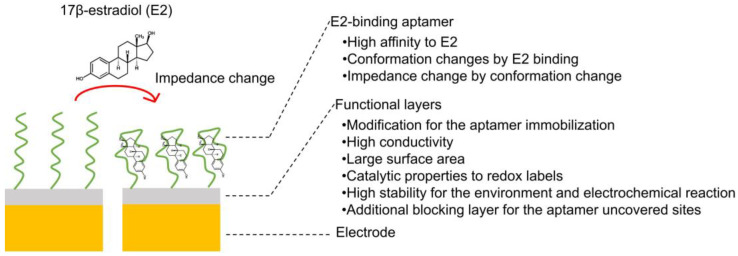
Simplified structure of estradiol aptasensors based on impedance change.

For better understanding, Figure 4 illustrates a simplified sensor structure and usual aptasensing mechanism. The details of the sensor structures and the fabrication of each component will be discussed in later sections.

### 5.2. Estradiol Aptasensing Mechanism

**Figure 5:**
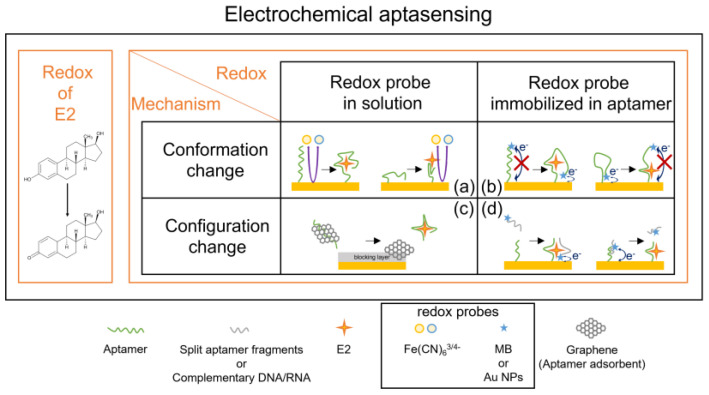
Classification of estradiol aptasensors based on the aptamer and redox probe behaviors. (**a**) Impedance change by the aptamer conformation change. Examples of impedance increase (left) and decrease (right) are shown. (**b**) Change in the redox current by the aptamer conformation change. Examples of the current increase (left) and current decrease (right) are shown. (**c**) Change in redox current by the configuration change in aptamer complex (formation or decomposition). An example of current increase by graphene current channel formation is shown. (**d**) Change in the redox current by the configuration change in the aptamer complex. Examples of split aptamer binding (left) and complementary DNA/RNA decomposition (right) are shown.

We have classified estradiol aptasensing mechanisms based on the aptamer conformation/configuration change and types of redox probe materials, as shown in Figure 5. In this section, we will discuss in detail the different types of estradiol aptasensing mechanisms. Among the four classes (a), (b), (c), and (d) in our literature survey, we could not find class (b) reports for estradiol sensing, but a schematic figure is inserted to illustrate how the sensing mechanisms are classified.

Detection of changes in impedance and/or in the redox current of Fe(CN)_6_^3−/4−^ is one of the most common methods in electrochemical E2 aptasensing (Figure 5a). DNA/RNA aptamers are negatively charged due to the phosphate groups in these oligo-nucleotides. The negatively charged aptamers provide a repulsion force to anions, such as Fe(CN)_6_^3−/4−^. When the E2 analytes bind to the aptamers, the conformation changes in the aptamers either disturb the ion transport or sometimes make the transport easier; the impedance may be increased [37,38,39] or decreased [40]. These sensors belong to class (a) in Figure 5.

Some authors utilized methylene blue (MB) as a redox probe instead of Fe(CN)_6_^3−/4−^. There may be an interaction between MB and ssDNA, specifically through the guanine bases of ssDNA [41].

Huang et. al. [42] designed ssDNA (single-stranded DNA), one end of which was guanine-rich, and the other end of which was complementary to an aptamer. This sensor belongs to class (d) in Figure 5. Without E2, the complementary aptamer (cDNA) was combined with an aptamer. As MB strongly adsorbed to the guanine-rich cDNA, the MB redox current was larger. With E2, aptamers were already bound, so that cDNA could not stick to the aptamers and the MB redox signal was decreased.

**Figure 6:**
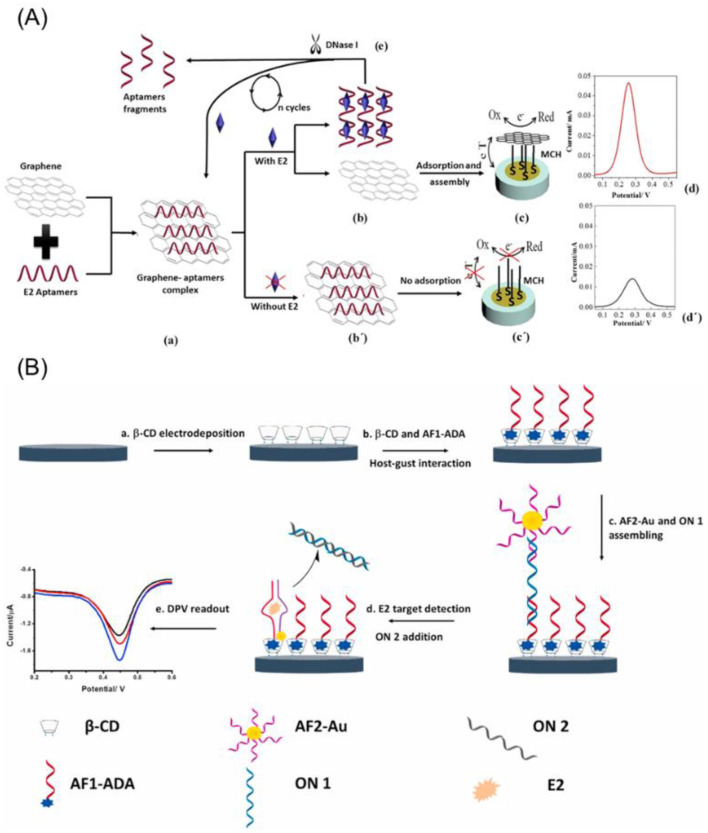
(**A**) Design and mechanism of E2 aptasensing technique based on graphene–aptamer complex and DNase 1 enzyme. Reprinted with permission from Ref. [44]. copyright © 2022, Elsevier, (**B**) Fabrication and mechanism of E2 aptasensor composed of aptamer fragments (AF1, AF2) and oligonucleotides (ON1, ON2). Reprinted with permission from Ref. [43]. copyright © 2022, Elsevier.

On the other hand, Zhu Chang et al. [43] utilized a more sophisticated technique, making use of two aptamer fragments (AF1 and AF2) and two complementary oligo-nucleotides (ON1 and ON2). Please see Figure 6 for the illustration. In the absence of E2, AF1 and AF2 are hybridized with ON1. When E2 analytes as well as ON2 are in solution, ON1 and ON2 can be hybridized, and AF1 and AF2 can be self-reconstructed during the E2 binding process. As a result, Au NPs bound with AF2 came closer to the electrode and the DPV signal was increased. This sensor belongs to class (d) in Figure 5, but Au NPs were used as redox probes instead of MB.

Graphene and aptamers may form a complex by their strong π–π interaction and M. Liu. et al. [44] utilized the graphene–aptamer complex formation in the E2 aptasensing. The authors used the MCH/Au electrode, and the self-assembled monolayer of MCH acted as an insulating barrier (Figure 6). Then, only a weak electrochemical current was observed without E2. However, in the presence of E2, the strong π–π stacking of graphene and aptamer might be broken, leading to the separation of the aptamers and the graphene. This is due to the stronger affinity and larger interaction force of the aptamers towards E2. Subsequently, the bare graphene could be accumulated on the MCH/Au electrode, resulting in a larger redox current (Figure 5c). Furthermore, the simultaneous addition of the DNase I enzyme initiated the cleavage of the aptamers and the release of E2. The released E2 analytes could further break the graphene–aptamer complex, which led to a cycling amplification of the electrochemical currents. The authors observed that the increment of the redox current was related to the E2 concentration.

### 5.3. Aptamer Immobilization Strategies

**Figure 7:**
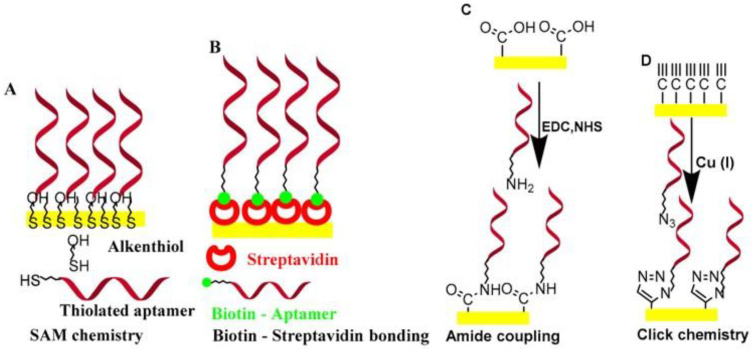
The common surface immobilization methods for aptamers. (**A**) SAM (self-assembled monolayer) chemistry, (**B**) biotin–streptavidin bonding, (**C**) amid coupling, and (**D**) click chemistry. Reprinted with permission from Ref. [46]. copyright © 2022, MDPI.

In electrochemical aptasensors, electrode materials not only have to be electrically conductive but also need to have high affinity and catalytic capacity for redox reaction on the electrode surface. However, high affinity is already warranted by the aptamers, and the strategies for the immobilization of the aptamers are more crucial for the choice of electrode materials. Depending on the physiochemical properties of the transducer material (metal, semiconductor, or polymer), an immobilization strategy of aptamers on the electrode surface will be required. In the case of E2 aptasensors, the aptamer can be attached to a wide range of surfaces including metal, metal oxide, and carbonaceous material. For aptasensing, one has to choose the proper functional form (-OH, amine, thiol, biotin, etc.) of the aptamers with available electrodes. Aptamer immobilization strategies play a vital role in the recognition of analytes by the electrode materials [45]. To date, numerous surface immobilization strategies have been developed, such as covalent bonding, electrostatic interaction, avidin–biotin interaction, and the self-assembled monolayer, for aptamer loading on the electrode surface. Several reports on the electrode material and estradiol immobilization can be found in the literature. Radi et al. [46] briefly discussed the most common aptamer surface immobilization methods (Figure 7). The electrode material and various binding strategies of E2-specific aptamers will be discussed in this section.

**Figure 8:**
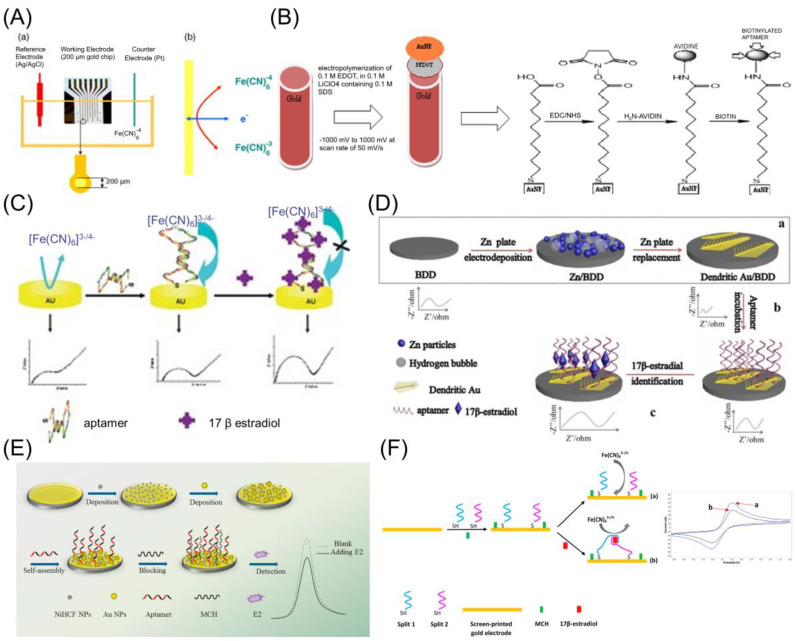
Electrochemical aptasensors based on gold electrode platforms for E2 detection. The gold-based aptasensor electrode platforms are the (**A**) gold/streptavidin/biotin–aptamer electrode. Reprinted with permission from Ref. [35] copyright © 2022, Elsevier, (**B**) gold/PEDOT/AuNPs/streptavidin/biotin–aptamer electrode. Reprinted with permission from Ref. [47] copyright © 2022, MDPI, (**C**) gold–aptamer electrode. Reprinted with permission from Ref. [38] copyright © 2022, The Royal Society of Chemistry, (**D**) dendritic gold/BDD electrode. Reprinted with permission from Ref. [37] copyright © 2022, Elsevier, (**E**) gold/nickel hexacyanoferrate/Au NPs electrode. Reprinted with permission from Ref. [48] copyright © 2022, Elsevier, (**F**) gold-split aptamer electrode. Reprinted with permission from Ref. [49] copyright © 2022, Elsevier. The gold–thiol bond aptamer immobilization strategy was implemented for gold-based electrodes.

As shown in Figure 8, for gold-based electrodes, the Au–S bond aptamer immobilization approach has been utilized. In particular, Kim et al. [35] used a well-known avidin–biotin interaction strategy to immobilize the selected DNA aptamer on a gold electrode. In the gold/streptavidin/biotin–aptamer electrode combination, the gold electrode surface is decorated with COOH groups to bind the amino group of streptavidin. On the streptavidin-modified gold electrode, biotinylated ssDNA of estradiol is attached by the interaction of the avidin–biotin interaction. A similar strategy of carbodiimide chemistry and the aptamer immobilized via the streptavidin–biotin interaction employed by Olowu, et al. [47] on electrochemically synthesized Poly(3,4-ethylenedioxylthiophene) (PEDOT) doped with gold nanoparticles (AuNPs) platform (Gold/PEDOT/AuNPs/streptavidin/biotin aptamer). In contrast, Lin et al. [38] applied a similar strategy but with fewer complications, by simply immersing the gold surface in a closed container containing a thiolated E2 aptamer for 2 h at 37 °C to form a self-assembled monolayer on the surface through the Au–S covalent bond.

The dendritic micro-structured gold electrodeposited B-doped diamond (BDD/Au) electrode surface was also immobilized in a closed container and incubated overnight at 37 °C with an E2 aptamer [37]. Owing to the benefits of gold nanoparticles, Fan et al. [48] electrodeposited Au nanoparticles (NPs) on NiHCF NPs, which acted as the matrix for anchoring the aptamer and improved the conductivity of the electrode. Before exposing the electrode to the aptamer, 0.2 mM Tris (2-carboxyethy1) phosphine was used to reduce the disulfide bond of the aptamer. Subsequently, the E2 aptamer was immobilized on the gold/NiHCF/Au NP-modified electrode incubated in 2.0 × 10^−6^ M aptamer solution for over 12 h. Nameghi et al. [49] designed an estradiol aptasensing platform by using estradiol-specific thiolated split aptamers. These split aptamers are immobilized on the surface of the gold electrode by the Au–S bond, whereas the mixture of split1 (250 nM) and split2 (250 nM) aptamers pretreated with TCEP was dropped onto the surface of the gold electrode to accomplish a binding reaction for 12 h at room temperature in a moisture-saturated environment.

**Figure 9:**
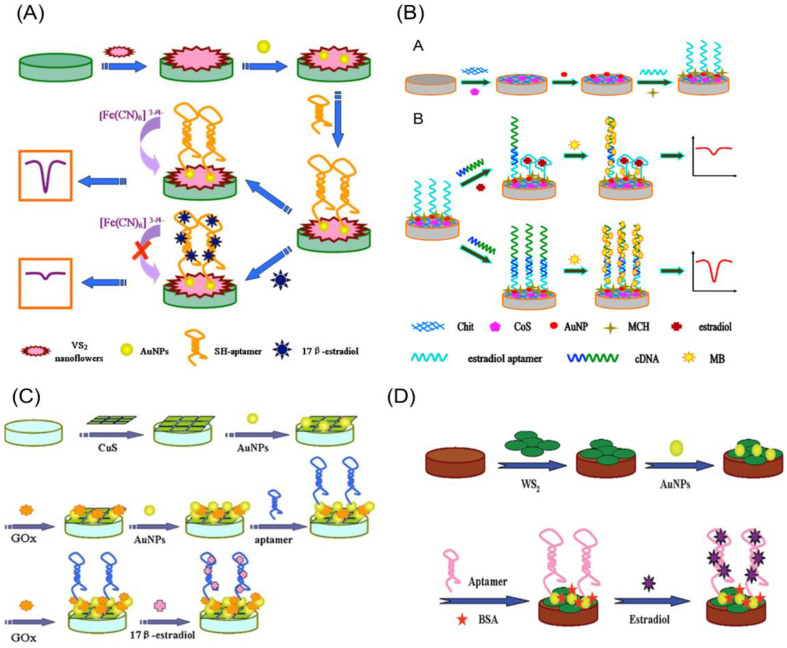
Electrochemical aptasensors based on semiconducting 2D-TMC and 2D-TMD electrode platforms for E2 detection. The semiconducting 2D-TMC and 2D-TMD aptasensor electrode platforms are (**A**) glassy carbon/VS_2_ nanoflower/gold–aptamer electrode. Reprinted with permission from Ref. [50]. copyright © 2022, Elsevier, (**B**) glassy carbon/CoS nanosheets/gold–aptamer electrode. Reprinted with permission from Ref. [42]. copyright © 2022, Elsevier, (**C**) glassy carbon/CuS/gold–aptamer electrode. Reprinted with permission from Ref. [52]. copyright © 2022, Springer-Verlag Wien, (**D**) glassy carbon/WS_2_/gold–aptamer electrode. Reprinted with permission from Ref. [51]. copyright © 2022, The Royal Society of Chemistry. The gold–thiol bond aptamer immobilization strategy was implemented for semiconducting 2D-TMC and 2D-TMD electrodes.

Huang et al. [42,50,51,52] implemented a similar strategy for semiconducting 2-dimensional transition metal chalcogenide (2D-TMC) and 2-dimensional transition metal dichalcogenide (2D-TMD) electrodes as shown in Figure 9. By simply incubating the electrode surface with an aptamer for an entire night, Huang et al. [50,51] were able to immobilize aptamers on glassy carbon/VS_2_ nanoflower/Au and glassy carbon/WS_2_ nanosheets/Au electrode surfaces through the Au–S interaction. In another study, Huang et al. [52] modified the glassy carbon electrode with a 2-dimensional CuS nanosheet/gold/glucose oxidase composite and embellished it with gold nanoparticles in order to facilitate the establishment of the Au–S bond for the loading of the E2-specific aptamer. Following this, Huang, et al. [42] achieved identical results of gold electrodeposition and the production of Au–S bonds using cobalt sulfate (CoS) nanosheets, and the aptamer was covalently conjugated by the Au–thiol bond (AuNPs/CoS/GCE).

**Figure 10:**
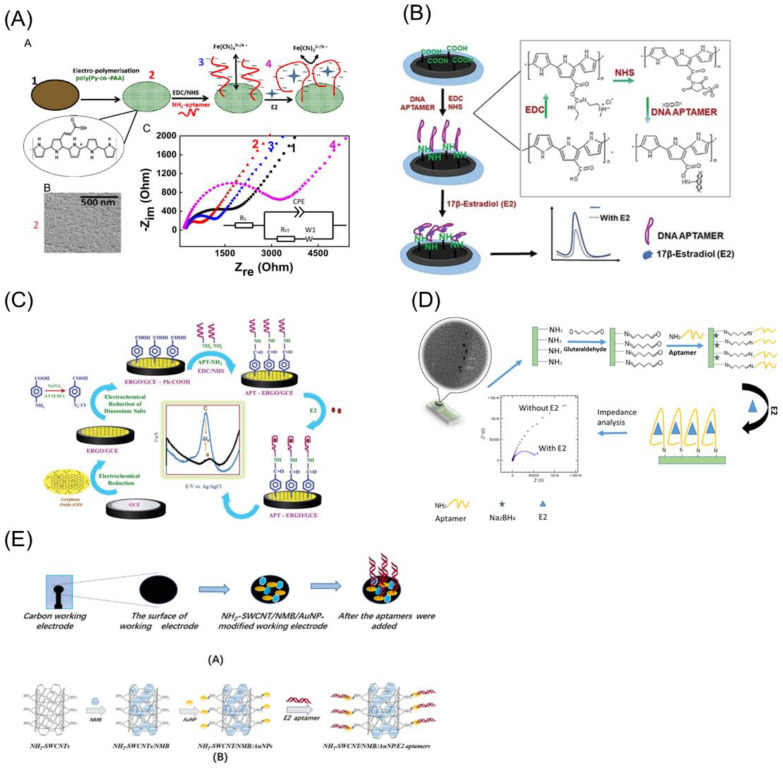
Electrochemical aptasensors based on carbon-based electrode platforms for E2 detection. The carbon-based aptasensor electrode platforms are the (**A**) glassy carbon/pyrrole (Py) and 3-pyrroleacrylic acid (PAA) (poly(Py-co-PAA)–aptamer electrodes. Reprinted with permission from Ref. [39]. copyright © 2022, Elsevier, (**B**) screen-printed carbon/poly(pyrrole-co-pyrrole-3-carboxylic acid (PPYPA)–aptamer. Reprinted with permission from Ref. [53]. copyright © 2022, Institute of Chemistry, Slovak Academy of Sciences, (**C**) glassy carbon/ERGO/diazonium salt (ClN_2_+–Ph–COOH)-aptamer electrode. Reprinted with permission from Ref. [13]. copyright © 2022, The Royal Society of Chemistry, (**D**) SPCE/CDs/glutaraldehyde–aptamer electrode. Reprinted with permission from Ref. [40]. copyright © 2022, MDPI, (**E**) Amine-functionalized single-walled carbon nanotube/new methylene blue/gold–aptamer electrode. Reprinted with permission from Ref. [54]. copyright © 2022, American Chemical Society. The carbodiimide interaction aptamer immobilization strategy was implemented for (**A**–**C**) through glutaraldehyde crosslinker for (**D**); through gold–thiol for (**E**) carbon-based electrodes.

For E2 aptasensors, Zhu et al. [39] designed an aptamer functionalized nanoporous conducting polymer electrode. To construct the sensor electrode, the poly(Py-co-PAA) copolymer was electropolymerized on a glassy carbon electrode, and the carboxylic acid groups on the copolymer’s surface were activated using EDC/NHS chemistry to promote the formation of an amide bond with the aptamer’s amine end. On the screen-printed carbon electrode, Rozi et al. [53] electrodeposited a layer of poly(pyrrole-co-pyrrole-3-carboxylic acid) (PPYPA), and an E2 aptamer was bound on the altered surface through a carbodiimide interaction. Where EDC-NHS serves as a coupling agent for amine functionalized aptamer immobilization through COOH-NH_2_ bonding, it was employed to activate the terminal carboxylic groups of the PPYPA film. The EDC/NHS coupling process with an NH-aptamer was described in detail by Rozi et al., as shown in Figure 10.

Working electrodes were printed with carbon ink on a piece of cellulose filter paper by Ming et al. [54] to construct an aptasensor. On the carbon working electrode, the nanocomposite solution containing the novel methylene blue, gold nanoparticles, and single-walled carbon nanotubes (NH_2_-SWCNTs) was dropped. This study used gold nanoparticles to bind NH_2_-SWCNTs via the Au–Amine interaction and E2 aptamer via the Au–thiol interaction.

Rather et al. [13] built a graphene-amplified electrochemical aptasensor in which graphene oxide is electrochemically reduced at a glassy carbon electrode and then electro-grafted by the electrochemical reduction of diazonium salt to obtain carboxylic groups on the electrode surface. Carbodiimide bonds were formed to immobilize the COOH group surface with an amine-functionalized E2 aptamer. The modified electrode was immersed in an amine-terminated aptamer solution containing EDC and NHS as a coupling agent for 3 h to allow the immobilization of the NH_2_-APT groups with the COOH groups on the electrode surface.

To improve detection efficiency, Zaid et al. [40] fabricated a screen-printed electrode with microwells and electrodeposited carbon dots on the electrode surface. This electrode surface was then treated with the homo-bifunctional cross-linker glutaraldehyde to bind the amine-functionalized aptamer. The glutaraldehyde covalently compelled the aptamer’s amine group and dramatically affected the electron movement, increasing the electron transfer resistance (R_ct_) value to approximately 73%.

Liu et al. [44] used graphene’s intrinsic properties of hydrophobicity and π-conjugation to immobilize the aptamer. The E2-specific ssDNA was adsorbed on graphene via π–π interactions. However, to load the aptamer on AuNP–Thi–CNTs nanocomposite electrodes, Liu et al. [55] used the potentiostatic method. The surface of the nanocomposite was partially passivated with MCH after deposition over a glassy carbon electrode, and the E2 aptamer was immobilized on AuNPs via the Au–S bond using a chronoamperometry technique.

Poly(β-Cyclodextrin) [poly(β-CD)]-modified electrode surfaces received extensive attention for electrochemical aptasensing due to their simultaneous existence of hydrophilic and hydrophobic characteristics [56,57]. Chang et al. [43] presented a disposable, laser-scribed graphene (LSGE) electrochemical sensing strip for the detection of E2 for use in portable sensing devices. Before using the LSGE strip, the sensing design and performance were tested on glassy carbon electrodes (GCE). On the surface of the GCE and LSGE, poly(β-cyclodextrin) (poly(β-CD)) was electropolymerized by CV. The amino terminated fragment (AF1) was covalently bonded with carboxyl acid functionalized adamantane using EDC/NHS chemistry (ADA). Furthermore, through host–guest interactions between the adamantane and poly(β-CD), this AF1-ADA is anchored to the (poly(β-CD))-modified electrode surface. Due to an inner hydrophobic cavity and an external hydrophilic surface, an intriguing property of -cyclodextrins allows them to selectively bind different host molecules in their cavities to form stable host–guest inclusion complexes [58]. β-CD can selectively bind a variety of guest molecules, such as ferrocene, m-methylbenzoic acid, and adamantine [59].

### 5.4. Blocking Layer Strategies

According to the conformational sensing mechanism, aptamer immobilization covers the electrode surface and insulates it from the redox probe, preventing it from reacting and producing the signal (a decrease in peak current). However, the aptamer does not cover the entire electrode surface area; it is idealistic to cover the entire electrode surface in the form of a monolayer, yet the formation of such a monolayer has not been reported in the literature. In order to fill the defects on the aptamer layer, an additional blocking layer is therefore required. According to Sharko et al. [60], the real electroactive surface area and electric double-layer capacitance change with the electrode blocking layer, so selecting the appropriate blocking agent is critical for the development of electrochemical aptasensors. Hence, the skill to stabilize DNA probes on electrode surfaces at optimal surface densities is important for the development of a broad range of DNA biosensors.

The electrodes in the electrochemical aptasensors for E2 are made of various nanomaterials, including gold, carbon, and others. The electrode surface with the specific aptamer layer is covered by an additional layer of blocking agents to fill the aptamer layer’s defects. Gold is one of the most frequently used in electrochemical estradiol aptasensing electrodes. Researchers mostly used MCH as a blocking agent to block the uncovered surface of gold during the immobilization of an E2-specific aptamer [35,42,44,54].

Since 1997, gold electrodes have been immobilized with DNA using the Hern and Tarlov method [61]. They achieved more precise control over the surface coverage of ssDNA on the electrode surface by forming mixed monolayers of thiol-terminated ssDNA and a spacer containing a thiol ligand, mercaptohexanol (MCH), to minimize nonspecific adsorption of single-stranded DNA. They specifically stated three reasons for choosing the MCH, which are summarized below. (1) ssDNA will not adsorb on the hydroxy-terminated surface of the MCH monolayer, (2) aqueous solubility, and (3) the six-carbon chain of MCH is not long enough to interfere with surface-bound DNA hybridization.

**Figure 11:**
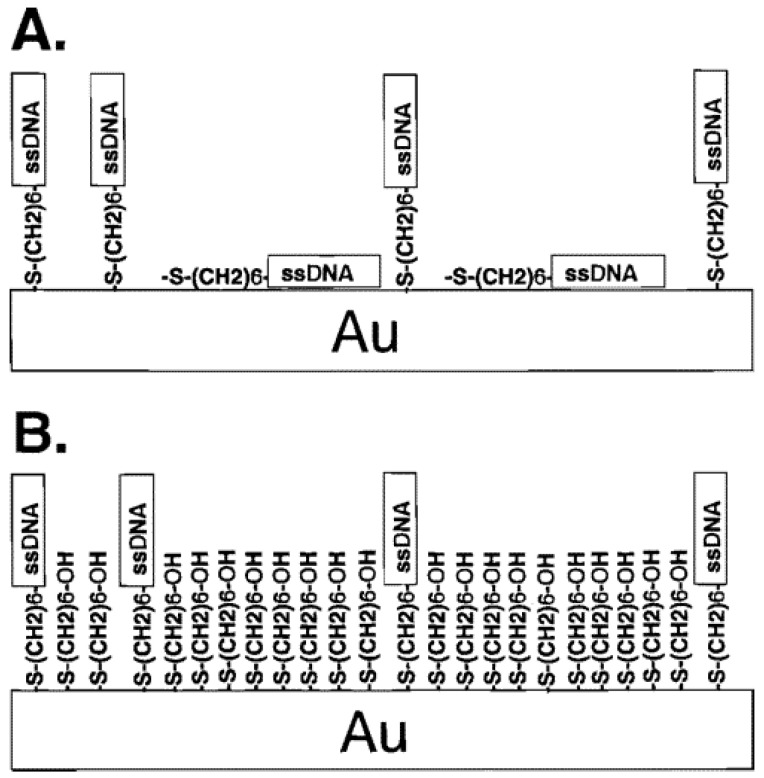
Schematic of (**A**) HS-ssDNA on Au and (**B**) both HS-ssDNA and MCH adsorbed on gold. Reprinted with permission from Ref. [61]. copyright © 2022, American Chemical Society.

Figure 11 depicts a schematic diagram of the detailed mechanism of monolayer formation [61]. In the absence of MCH (part A), the authors hypothesized that the nitrogen-containing nucleotide bases of HS-ssDNA (5′-HS-(CH_2_)_6_-CAC GAC GTT GTA AAA CGA CGG CCA G-3′) and the sulfur atoms in the thiol group interact with the surface. On the other hand, it was predicted that the nucleotide bases would not interact with the surface in the presence of thiolated-MCH and that HS-ssDNA molecules would instead adsorb on the surface through Au–S interaction. Similar to this, Keighley et al. [62] described a technique for controlling DNA surface density by co-immobilizing MCH and thiol-modified DNA simultaneously.

Even though the MCH is one of the most well-known blocking layer materials, there are several other materials reports. For example, Huang et al. [51] incubated the modified electrode with bovine serum albumin (BSA) and then washed it with phosphate-buffered saline (PBS) to obstruct the exposed surface of the glassy carbon/WS_2_ nanosheets/Au surface. According to Haung et al. [52], glucose oxidase works as both a signal amplifier and a signal blocker in the electrode configuration of GOx/aptamer/AuNPs/GOx/AuNPs/CuS/GCE. In addition, the unbound aldehyde functional groups of glutaraldehyde crosslinker on the aptamer-modified SPCE passivated by reacting them with the sodium borohydride (Na_2_BH_4_) [40]. While immobilizing the aptamer on a gold electrode via the streptavidin–biotin interaction, ethanolamine is used as a blocking agent to block the remaining -COOH groups after streptavidin modification [35].

### 5.5. Electrode Material and Estradiol Aptasensing Performance

**Figure 12:**
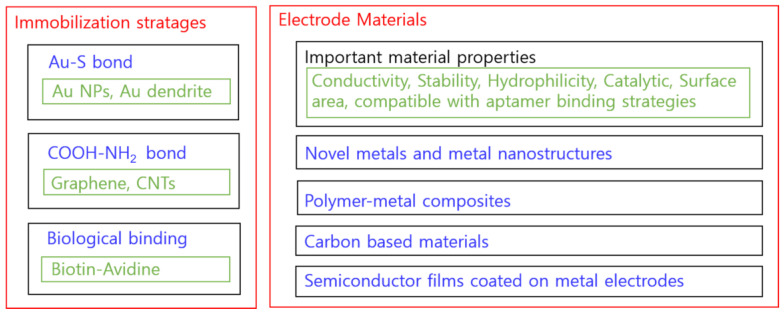
Immobilization strategies and electrode materials.

In this section, we discuss the electrode materials and aptasensing performance, such as the detection limits, reproducibility, and selectivity. In the previous section, we explained functional groups and aptamer immobilization. The bonding of functional groups is known depending on the type of electrode material (e.g., Au–S in gold, -COOH and -NH_2_ bond in carbon, etc.). The electrode material not only affects the immobilization of the aptamer, but also the conductivity, surface morphology, and catalytic effect of the material itself, greatly affecting the sensor properties (Figure 12). In this section, we describe electrode materials in the order of Au, semiconductor, carbon, and polymers.

#### 5.5.1. Metal Electrodes

Despite the availability of other metal electrodes, gold is commonly preferred because a thiolated aptamer can be immobilized using a simple physisorption procedure [63].

In 2007, Kim et al. [35] designed an electrochemical estradiol aptasensor using a gold electrode chip. The aptasensor exhibited an E2 detection limit of 0.1 × 10^−9^ M with a linear range from 1 to 0.01 × 10^−9^ M. They also demonstrated the reproducibility of the aptasensor, and the standard deviation of the electrochemical current was within 17.4%. Since then, the E2 aptamer sequence has been extensively studied.

Lin et al. [38] developed an electrochemical estradiol aptasensor using a gold electrode. Their aptasensor demonstrated good stability and selectivity for E2 with an improved detection limit of 2.0 × 10^−12^ M in a linear range from 1.0 × 10^−8^ to 1.0 × 10^−11^ M and was employed to identify E2 in human urine as well. However, the authors mentioned that incubation time plays a crucial role in sensor performance. They noticed a sudden change in the impedance after 4 h of incubation time; the longer incubation time required may be due to the slow binding of E2 with the aptamer. This longer incubation time limits the proposed sensor for real-time analyte sensing. In 2019, Nameghi et al. [49] fabricated an E2 aptasensor having an enhanced detection limit of 0.5 × 10^−12^ M with a shorter incubation period of 30 min by employing split aptamers on the screen-printed gold electrode. The aptasensor showed selectivity between E3, progesterone (PRG), dibutyl phthalate (DBP), BSA, testosterone, atrazine, and BPA. The sensor showed a detection limit of 0.5 × 10^−12^ M in tap water and 0.7 × 10^−12^ M in the milk sample.

Fan et al. [48] used NiHCF NPs as a signal probe to avoid the complex aptamer labeling (MB) or adding an extra probe Fe(CN)_6_^3−/4−^ into the test system. They constructed an E2 aptasensor by depositing AuNPs on nickel hexacyanoferrate nanoparticles (NiHCF NPs) to anchor the aptamer and increase the electrode’s conductivity. The prepared aptasensor performed well, with a detection limit of 0.8 × 10^−12^ M over a linear range from 1 × 10^−12^ to 6 × 10^−10^ M. The selectivity of the aptasensor was investigated in different endocrine disruptors, such as ethinylestradiol, BPA, E3, polychlorinated biphenyl (PCB 101), diethyl phthalate, 4-nonyl phenol, and atrazine. The E2 detection feasibility of the aptasensor was evaluated in the municipal wastewater samples.

The optical, electrical, and catalytic activities of the gold nanostructures depend on their morphology. The different morphologies of nanoparticles with specific surface atomic arrangements influence their catalytic active sites and, thus, their catalytic behavior [64]. Dendritic nanostructures have received a lot of attention among the many morphologies due to their prospective uses in surface-enhanced Raman spectroscopy (SERS) sensing, catalysis, and electrocatalysis [65]. Ke et al. [37] fabricated a hierarchical dendritic gold microstructure on a boron-doped diamond (BDD) surface (Au/BDD). The dominant crystallographic plane (111) of the dendritic gold structure favors a rapid electron transfer rate and resists unwanted PBS adsorption, resulting in a lower background signal and high catalytic activity. The dendritic gold also provides a large surface area with many active sites for aptamer binding. The aptasensor has a low detection limit of 5 × 10^−15^ M and selectivity in E3, BPA, nonyl phenol, diethyl phthalate, resorcinol, and atrazine. The detection of E2 in the wastewater samples demonstrated the reliability and potential of the aptasensor.

#### 5.5.2. Semiconducting Electrodes

In addition to the dendritic gold, many researchers focused on 2D-TMC and 2D-TMD nanomaterials in the development of electrochemical sensors. The layered structure of 2D-TMC and 2D-TMD offers fast electron transfer due to active sites on their edges and their large surface area can act as a host for various nanomaterials. The decoration of noble metals on 2D-TMC and 2D-TMD nanosheets has been considered an efficient way to improve the electrochemical sensor’s performance [66]. Huang and colleagues made efforts to develop the electrochemical E2 aptasensors by incorporating the noble gold nanoparticles with different 2D transition metal chalcogenides, such as vanadium disulfide (VS_2_) [50], tungsten disulfide (WS_2_) [51], cobalt sulfide (CoS) [42], and copper sulfide (CuS) [52]. Even though semiconducting materials are inappropriate as sensing electrodes, Huang used these materials with AuNPs because 2D nanosheets significantly increase the electrode’s active surface area, while the AuNP coating on the nanosheets encourages electron transfer and improves the aptamer loading, which results in high sensitivity.

The VS_2_-based aptasensor was constructed by immobilizing aptamer on the gold-decorated surface of the VS_2_ nanoflower-modified glassy carbon electrode. The high loading ability of VS_2_ nanoflowers for AuNPs, as well as the unique properties of VS_2_, DPV determined E2 with a detection limit of 1.0 × 10^−12^ M with logarithm concentrations ranging from 1.0 × 10^−11^ to 1.0 × 10^−8^ M. Additionally, the aptasensor was successfully applied for the determination of E2 in urine samples. Among structurally similar organic chemicals, naphthalene and 1-aminoanthraquinone, the aptasensor demonstrated significant reproducibility and selectivity [50]. Similarly, the layered WS_2_ nanosheets/AuNPs-based aptasensor showed high sensitivity with a detection limit of 2.0 × 10^−12^ M for E2 and selectivity between naphthalene and 1-aminoanthraquinone. The aptasensor was implemented for the detection of E2 in serum and water samples [51].

In another study, Huang et al. [42] designed an aptasensor based on AuNPs/CoS nanosheets/GCE using methylene blue (MB) as an indicator and guanine-rich complementary DNA (cDNA) as a signal amplifier. Where cDNA hybridizes with an aptamer that is unbound with target E2, selectivity is enhanced and the redox signal of MB is amplified. They achieved a low detection limit of 7.0 × 10^−13^ M for E2 with a dynamic concentration range from 1.0 × 10^−9^ to 1.0 × 10^−12^ M. Among 1-aminoanthraquinone, naphthalene, polychlorinated biphenyl, BPA, phthalic acid ester, testosterone, and cholesterol, the aptasensor demonstrated good stability and selectivity for E2. Further, a novel electrochemical aptasensor was developed by utilizing the glucose oxidase (GOx) enzyme as a signal amplifier with AuNP-modified CuS. This combination accelerated the electron transfer and enhanced the detection limit up to 60 × 10^−15^ M with a linear range from 5.0 × 10^−13^ to 5.0 × 10^−9^ M [52]. Both of the developed aptasensors were employed to detect E2 in urine samples.

#### 5.5.3. Carbon-Based Electrodes

Carbon-based nanomaterials such as graphene, graphene oxide, and carbon nanotubes have attracted great attention in the last decade. Extraordinary properties, such as conductivity, large surface-to-volume ratio, biocompatibility, ease of synthesis, and functionalization abilities make them good candidates for biosensors. These materials also have a large potential window, excellent electrocatalytic activity, and are inexpensive [67].

Rather et al. [13] constructed a novel graphene-amplified femto-sensitive aptasensor for E2 sensing. The outstanding 0.5 × 10^−15^ M detection limit was achieved through the synergistic effect of the E2 aptamer and graphene platform. The oxidation current peaks were proportional to E2 over the concentration range from 1.0 × 10^−15^ to 0.23 × 10^−9^ M. The designed aptasensor demonstrated E2 selectivity in both estrogen and testosterone. The practical application was tested in real wastewater and pharmaceutical samples. In this sensor, rGO served to anchor NH_2_ due to the COOH present on the surface, while at the same time providing a large surface area by geometric folding, wrinkling, and rippling of graphene sheet flakes.

As discussed in the previous section, Liu et al. [44] fabricated sensors using graphene–aptamer composites. Unlike other cases, the composites were not directly deposited on the electrode surface. When E2 molecules were present in the solution, aptamers were bound to the E2 molecules and were separated from the graphene. Then, the graphene was adsorbed on the electrode surface, and the redox currents were greatly increased. Using graphene’s inherent properties and DNase I cycling amplification, this aptasensor achieved a low detection limit of 50 × 10^−15^ M with a linear range from 0.07 × 10^−12^ M to 10 × 10^−12^ M. The authors also reported an E2 aptamer incubation time of less than 1 min, satisfactory stability, reproducibility, and high selectivity with coexisting E3, BPA, nolyl phenol, polychlorinated biphenyl (PCB), and atrazine. The developed aptasensing system was further applied to detect E2 in the tap water samples.

Similarly, Chang et al. [43] constructed a single-use, disposable, split aptamer-based laser-scribed graphene electrochemical (LSGE) sensing strip. With a wide dynamic detection range from 10^−15^ M to 10^−9^ M, a low detection limit of 0.7 × 10^−15^ M was achieved for the GCE electrode platform; however, a comparable 63.1 × 10^−15^ M low detection limit was found for the disposable LSGE electrode. The highly porous structure of LSGE is non-favorable for selectivity due to the nonspecific adsorption of interfering agents, whereas the GCE shows better selectivity for glucose, BPA, PRG, and BSA. The disposable LSGE strip showed the feasibility of the developed E2 sensor in a real milk sample with the portable device.

Liu et al. [55] prepared the ratiometric electrochemical aptasensor (REAS) for E2 detection by decorating AuNPs on the surface of thionine (Thi)-loaded CNTs. In this prepared AuNP–Thi–CNT nanocomposite electrode, thionine serves to immobilize AuNPs through Au–N bonds, and also serves as a reference signal indicator in ratiometric electrochemical measurements. The E2 aptamer complex partially blocks the electron transfer channel when an E2 target is present, which raises the oxidation signal of E2 molecules in DPV measurements while simultaneously decreasing the oxidation signal of the thionine. With a linear range from 12 × 10^−12^ M to 60 × 10^−9^ M for E2 concentration, the sensor achieved a 1.5 × 10^−12^ M lower detection limit by comparing the peak current ratio of thionine and E2 using a dual signal strategy. The REAS showed good specificity among ascorbic acid, dopamine, uric acid, L-lysine, L-cysteine, L- tyrosine, hypoxanthine, PRG, and cholesterol. The aptasensor was successfully applied to the detection of E2 in human serum samples collected from females, where E2 was spiked into diluted serum samples.

Ming et al. [54] fabricated a folding paper-based aptasensor electrode coated with amine-functionalized single-walled carbon nanotube/new methylene blue/AuNPs. The paper was a biocompatible porous cellulose fiber web. The large surface area and porous structure of papers allowed a large number of attachment points for nanoassemblies, amplifying the detection signal. Since materials such as CNTs and graphene can be adsorbed well to other materials by pi–pi interactions, NMB was immobilized on CNTs without additional treatment. The detection limit of this sensor was approximately 5 pg mL^−1^, with a linear range between 10 pg mL^−1^ and 500 ng mL^−1^. The experimental results showed that E2 detection by this sensor had high selectivity when E2 was mixed with follicle-stimulating hormone (FSH), luteinizing hormone (LH), glutamic acid (Glu), ascorbic acid (AA), uric acid (UA), neuron-specific enolase (NSE), or carcinoembryonic antigen (CEA). Experiments to detect E2 in clinical serum samples were also carried out to evaluate the capability of the aptasensor in real samples and the results were comparable to those obtained using a commercially available electrochemical luminescence apparatus.

The nanometer-sized carbon quantum dots have been studied more as electrocatalysts rather than as an electrode material in electrochemical sensors. Zaid et al. [40] fabricated a high-sensitive aptasensor using carbon dots as electrode surface modifiers synthesized by a simple hydrothermal method. The developed aptasensor showed an excellent low detection limit of 0.5 × 10^−12^ M for E2 detection, with a linear range from 1 × 10^−7^ to 1 × 10^−12^ M. Moreover, the aptasensor showed excellent specificity in the complex water system with three different matrixes of E3, PRG, and BPA, and it was also successfully applied to detect E2 in real environmental samples.

#### 5.5.4. Polymer Electrodes

Conducting polymers (CPs) can be easily fabricated via electro-polymerization and have excellent properties for electrochemistry, such as good hydrophilicity, nanoporous morphology, easy control of the surface area, good adhesion of aptamers, stability in aqueous solution, and high electrical conductivity. CP is also known to have electrocatalytic activity. Due to these characteristics, many studies have been conducted on electrochemical sensors using CP [68].

Compared to metal nanoparticles or conducting polymers used alone, conducting polymer–metal nanocomposites often exhibited improved properties, such as excellent conductivity, stability, catalytic activity, effective surface area, and other synergetic effects [69,70]. The Poly(3,4-ethylenedioxythiopene) (PEDOT)–gold nanocomposite was studied for electrochemical E2 aptasensing and was reported to have a catalytic effect on the Fe(CN)_6_^3−/4−^ redox probe [47]. The catalytic effect improved the current signal and sensitivity. The linear range and detection limit were 0.1 × 10^−9^ to 100 × 10^−9^ M and 0.02 × 10^−9^ M. The stability and reproducibility of the manufactured aptasensor were confirmed up to six times by square wave voltammetry, and after 15 days of storage, the electrochemical response was 75.53 % of its initial response.

Porous conducting polymers are also good candidates for electrochemical sensing applications due to their larger specific surface area and more active sites [71]. For electrochemical E2 aptasensing, poly(Py-co-PAA) nanoporous copolymer was used [39]. The aptamer folding around a neutral E2 molecule caused a redistribution of charge at the electrode interface and a nanoporous conducting polymer electrode surface amplified this effect. The authors mentioned that coupling of a shorter 35-mer aptamer leads to a smaller EIS response than that for a 75-mer aptamer. Although a shorter aptamer exhibited improved affinity to E2 [72], the EIS signal was weaker for a shorter aptamer. The E2 sensor demonstrated reproducibility and selectivity against a non-steroidal bisphenol-A estrogenic compound and structurally related steroidal hormone progesterone. Moreover, they also tested in urine and tap water. The linear range and detection limit were 10^−15^ to 10^−6^ M and 10^−15^ M in their sensing device.

There were several efforts not only for nanocomposite structural development but also for improving conducting polymer properties for E2 aptasensing. The conjugate double bond structure of polypyrrole (PPY) offered an efficient flow of electrons in the polymer chain resulting in a higher conductivity than other conducting polymers [73]. The high conductivity and easy electro-polymerization of PPY made it suitable for electrochemical biosensors. On the other hand, Li et al. [74] claimed that the hydrophobicity of PPY influenced impedimetric DNA and protein sensing. To overcome this limitation, Truong et al. [75] added the hydrophilic pyrrole-3-carboxylic acid (PA) monomer to PPY for sensing the human chorionic gonadotropin. Henceforth, Rozi et al. [53] combined PA with PPY in order to achieve hydrophilicity, conductivity, and the COOH group for covalent binding of the aptamer. The largest current change in E2 was observed for the PPY:PA copolymer with a 3:1 ratio, with a detection limit of 30 × 10^−6^ M and a linear concentration range from 0 to 100 × 10^−6^ M, in comparison to 2:1 and 1:1 of the copolymer’s ratio.

Utilizing nanomaterials such as graphene oxide (GO) as a binder, there was another effort to improve the electrochemical activity, mechanical stability, and other material properties of PEDOT-based polymers. Zhao et al. [76] used a PEDOT–GO composite to fabricate a highly sensitive and stable aptasensor. They decorated Au@Pt nanocrystals and aptamers over electrodeposited PEDOT–GO. The aptasensor was passivated with MCH. After measurement of 10^−9^ M E2 repeated every 2 days for a total of 12 days at 4 °C, the stability rates were calculated as above 88.2% after 11 days. The linear range and detection limit were from 10^−16^ to 10^−12^ M and 8 × 10^−17^ M. The developed electrochemical aptasensor showed excellent specificity towards E2 among the structurally similar compounds BPA, estrone, E3, and ethinylestradiol. The aptasensor was employed to detect E2 in real samples of tap water and lake water, as well as human serum collected from healthy females in order to assess the aptasensor applicability. The developed aptasensing results were in good agreement with HPLC’s results.

## 6. Summary and Discussion

Estrogens in the environment represent a substantial risk to health and reproduction in numerous species, including humans. Therefore, the measurement of estrogens in the environmental context is of substantial interest. In our literature survey, we found E2 electrochemical aptasensing in urine [38,39,42,49,50,52] and in tap water [39,44,49]. Although the E2 detection in the serum of various species is very important in health-care, very few reports are available [51,54,55,76]. Further environmental investigations together with the matrix effects (interference effects from various materials) would be beneficial.

For E2 electrochemical sensing without using aptamers, direct oxidation of E2 was often monitored, but E2 electrochemical aptasensing was mostly based on conformation change in aptamers, utilizing the redox probes in solution. For E2 electrochemical aptasensing, choosing proper aptamers and aptamer immobilization to electrodes are necessary. We categorized similar chemical bonds for aptamer immobilization and discussed various immobilization strategies.

The DNA sequences of various E2 aptamers, which have been utilized up to now, are listed in Supplementary Table S1. As the sensing performance depends on various factors, including aptamer immobilization and electrode materials, it is difficult to directly compare the performances of various E2 aptamers. For the development of a new E2 aptamer sequence, molecular docking simulations or molecular dynamics simulations may be useful [77].

The electrode materials are not only important for the immobilization of the aptamers, but also influence the device performance due to their conductivity, surface morphology, and catalytic effect. We grouped the available literature by the type of electrode materials and discussed their performances. Ideally, immobilized aptamers should cover the whole electrode surface, which is very difficult to achieve. In order to fill the surface area that is not covered by the aptamers, an additional blocking layer was necessary.

## 7. Concluding Remarks

In the past few decades of biosensor research, aptamers have attracted significant attention as the next-generation target receptors that can replace antibody functions. 17β-estradiol is one of the endocrine-disrupting chemicals because it can interfere with normal endocrine functions in humans and wild animals. Research in the field of electrochemical biosensors for estrogen detection is very active and significant progress has been made. In this review, we categorized estradiol aptasensing mechanisms, aptamer binding strategies, electrode materials, and blocking layer strategies, which have been implemented until now. Although the detection limits of 17β-estradiol aptasensors have been greatly improved, most aptasensors have still been proof-of-concept demonstrations until recently. For commercial applications, more efforts should be directed to improve reproducibility, reduce false signals, and develop miniaturized handheld kits as well as automation programs for real-time electrochemical estradiol detection.

## Figures and Tables

**Table 1:** Electrochemical estradiol sensors based on direct estradiol oxidation.

Electrode	Linear Range (M)	Limit of Detection (M)	Measurement Technique	Reference
poly(NPEDMA)/Au	1 × 10^−7^–8 × 10^−7^	6.86 × 10^−7^	CV	[27]
rGO-DHP/GCE	4 × 10^−7^–2 × 10^−5^	7.7 × 10^−8^	CV, EIS, LSV	[28]
rGO/CuTthP/GCE	1 × 10^−8^–1 × 10^−6^	5.4 × 10^−9^	CV, DPV	[29]
GQDs-PSSA/GO/GCE	1 × 10^−9^–6 × 10^−6^	2.3 × 10^−10^	CV, DPV	[30]
MWCNTs/Au NPs/GCE	1 × 10^−6^–2 × 10^−5^	7.0 × 10^−8^	CV, LSV	[31]
Carbon dots-PANI/GCE	1 × 10^−9^–1 × 10^−4^	4.3 × 10^−8^	CV, LSV	[32]

## Data Availability

Not applicable.

## Supplementary Materials

The following supporting information can be downloaded at https://www.mdpi.com/article/10.3390/bios12121117/s1. Table S1: Electrode materials and aptamer sequences. https://drive.google.com/drive/folders/1aY4x8fNV-F0DSrKEA9WX1B5JFJOG7eAX?usp=sharing, accessed on 21 November 2022. References [13,35,37,38,39,40,42,43,47,48,49,50,51,52,53,54,55,76] have been cited in the Supplementary Materials.
