# Supplementary material for: Role of Estradiol Hormone in Human Life and Electrochemical Aptasensing of 17β-Estradiol: A Review
Source: Biosensors (Basel). 2022 Dec 2;12(12):1117. doi: 10.3390/bios12121117 (PMC9776368; doi:10.3390/bios12121117)
Supplement: Supplementary file 1 [file biosensors-12-01117-s001.zip › biosensors-2023436-supplementary.pdf]

biosensors

MDPI

Supporting Information

Role of Estradiol Hormone in Human Life and Electrochemical Aptasensing of 17β-Estradiol: A Review

P. P. Waifalkar [1,2], Daegwon Noh [1,2], Poorva Derashri [3], Sagar Barage [3,4] and Eunsoon Oh [1,2,*]

[1] Department of Physics, Chungnam National University, Daejeon 34134, Republic of Korea
[2] Institute of QuanTable 34134, Republic of Korea
[3] Amity Institute of Biotechnology, Amity University, Maharashtra, Mumbai-Pune Expressway, Panvel, Maharashtra 410206, India
[4] Centre for Computational Biology and Translational Research, Amity University, Maharashtra, Mumbai-Pune Expressway, Panvel, Maharashtra 410206, India
* Correspondence: esoh@cnu.ac.kr; Tel.: +82-10-6349-5453

Table S1. Electrode materials, aptamer sequences, selectivity in different matrix and environmental sample measurements.

Electrode	Estradiol Specific Aptamer Sequence	Selectivity in a Matrix (Interference Effects from Various Materials)	Environmental Sample Measurements	References
Au/streptavidin/biotin	5'-Biotin-GCTTCCAGCTTATTGAATTACACGCAGAGG-GTAGCGGCTCTGCGCATTCAATTGCTGCGCGCTGAAG-CGCGGAAGC-3'	2-methoxynaphthalene, 1-aminoanthraquinone	N/A	(Kim et al., 2007) [34]

Electrode	Estradiol Specific Aptamer Sequence	Selectivity in a Matrix (Interference Effects from Various Materials)	Environmental Sample Measurements	References
PEDOT/AuNP /streptavidin	5′-Biotin-GCTTCCAGCTTATTGAATTACACGCAGAGGGTAGCGGCTCT GCGC ATTCAATTGCTGCGCGCTGAAGCGCGGAAGC-3′	17α-ethynlestradiol, estrone, naphthalene	N/A	(Olowu et al., 2010) [46]
Au	5'-SH-GCTTCCAGCTTATTGAATTACACGCAGAGGGTAGCGGCTCT GCGCATTCAATTGCTGCGCGCTGAAGCGCG GAAGC-3'	2-methoxynaphthalene, 1-aminoanthraquinone	Urine	(Lin et al., 2012) [37]
GCE/VS2/Au	5'-SH-(CH2)$_6$-TTT TTT TTT T GCT TCC AGC TTA TTG AAT TAC ACG CAG AGG GTA GCG GCT CTG CGC ATT CAA TTG CTG CGC GCT GAA GCG CGG AAG C-3'	Naphthalene, 1-aminoanthraquinone	Urine	(Huang et al., 2014a) [50]
BDD/Au	5′-SHGCTTCCAGCTTATTGAATTACACGCAGAGGGTAGCGGCT CT- GCGCATTCAATTGCTGCGCGCTGAAGCGCGGAAGC-3'	Estriol, bisphenol A, nonyl phenol, diethyl phthalate, resorcinol, and atrazine	Wastewater	(Ke et al., 2014) [36]
GCE/WS2 /Au	5′-SH-(CH_2)$_6$-TTT TTT TTT T GCT TCC AGC TTA TTG AAT TAC ACG CAG AGG GTA GCG GCT CTG CGC ATT CAA TTG CTG CGC GCT GAA GCG CGG AAG C-3′	Naphthalene, 1-aminoanthraquinone	Serum and Water	(Huang et al., 2014) [51]
GCE/CuS/Au/ GO/Au	5′-SH-(CH_2)$_6$-TTT TTT TTT T GCT TCC AGC TTA TTG AAT TAC ACG CAG AGG GTA GCG GCT CTG CGC ATT CAATTG CTG CGC GCT GAA GCG CGG AAG C-3′	Bisphenol A, 1-aminoanthraquinone and naphthalene	Urine	(Huang et al., 2015a) [52]

Electrode	Estradiol Specific Aptamer Sequence	Selectivity in a Matrix (Interference Effects from Various Materials)	Environmental Sample Measurements	References
GCE/AuNp/ CoS	5'-SH-$(CH_2)_6$-TTT TTT TTT T GCT TCC AGC TTA TTG AAT TAC ACG CAG AGG GTA GCG GCT CTG CGC ATT CAA TTG CTG CGC GCT GAA GCG CGG AAG C-3'<br>cDNA: 5'-GGA GGA GGA GGA GGA GGA GGA GGA GGA GGA GGA GGA GGA G G CTT CCG CGC TTC AGC GCG CAG CAA-3'	1-aminoanthraquinone, naphthalene, polychlorinated biphenyl, bisphenol A, phthalic acid ester, testosterone, and cholesterol	Urine	(Huang et al., 2015b) [42]
Au/NiHCF/Au	5′-SH-$(CH_2)_6$-GCT-TCC-AGC-TTA-TTG-AAT-TAC-ACG-CAG-AGG-GTA-GCG-GCT-CTG-CGC-ATT-CAA-TTG-CTG-CGC-GCT-GAA-GCG-CGG-AAG-C-3′ ;<br>Random DNA sequence: 5′-(SH)-$(CH_2)6$-CTG-ACA-CCA-TAT-TAT-GAA-GA-3′	Ethinylestradiol, bisphenol A, estriol, polychlorinated biphenyl (PCB 101), diethyl phthalate, 4-nonyl phenol, and atrazine	Municipal wastewater	(Fan et al., 2015) [48]
GCE/poly(Py-co-PAA)	75-mer E2 aptamer:<br>$NH_2(CH_2)_6$-ATACGAGCTTGTTCAATACGAAGGGATGCCGTTTGGGCCCAAGTTCGGCATAGTGTGGTGATAGTAAGAGCAATC<br>35-mer E2 aptamer:<br>$NH_2(CH_2)_6$-AAGGGATGCCGTTTGGGCCCAAGTTCGGCATAGTG<br>Adenosine aptamer:<br>$NH_2(CH_2)_6$-ACCTGGGGGAGTATTGCGGAGGAAGGT	Bisphenol-A, progesterone	Urine and tap water	(Zhu et al., 2015) [39]

Electrode	Estradiol Specific Aptamer Sequence	Selectivity in a Matrix (Interference Effects from Various Materials)	Environmental Sample Measurements	References
	Non-specific ssDNA as control in urine detection: $NH_2(CH_2)_6$-AGGTATTGATCCGCCCGGCTAGCATTTGTGGTCTCCAGGGGTACTCCCC			
GCE/ERGO	38-mer amine-functionalized ssDNA (NH_2–APT)	Estrogen and testosterone	Wastewater and pharmaceutical sample	(Rather et al., 2018) [13]
Au/MCH/ Graphene	5′-GCTTCCAGCTTATTGAATTACACGCAGAGGGTAGCGGCTCTGCGCAT TCAATTGCTGCGCGCTGAAGCGCGGAAGC-3′	Estriol, bisphenol A, nolyl phenol, polychlorinated biphenyl and atrazine	Tap water	(Liu et al., 2019) [44]
Screen printed gold electrode	5′-Thiol-TTTTTTTTTTTTTTTGCTTCCAGCTTATTGAATTACACGCAGAGGGTA-3′ (split1);<br>5′-GCGGCTCTGCGCATTCAATTGCTGCGCGCTGAAGCGCGGAAGCTTTTTTTTTTTT-Thiol-3′ (split2)	Estriol, progesterone, dibutyl phthalate, bovine serum albumin, testosterone, atrazine, and bisphenol A	Tap water and milk	(Nameghi et al., 2019) [49]
Carbon/NH_2-SWCNT/NMB /AuNP	5′-SH-$(CH_2)_6$-GCT-TCC-AGC-TTA-TTG-AAT-TAC-ACG-CAG-AGG-GTA-GCG-GCT-CTG-CGC- ATT-CAA-TTG-CTG-CGC-GCT-GAA-GCG-CGGAAG-C-3′	Follicle-stimulating hormone, luteinizing hormone, glutamic acid, ascorbic acid, uric acid, neuron-specific	Serum	(Ming et al., 2019) [54]

Electrode	Estradiol Specific Aptamer Sequence	Selectivity in a Matrix (Interference Effects from Various Materials)	Environmental Sample Measurements	References
		enolase, or carcinoembryonic antigen		
AuNP/thionine/MCNTs	5′-SH-$(CH_2)_6$-GCTTCCAG CTTATTGAATTACACGCAGAGGGTA GCGGCTCTGCGCATTCAATTGCTGCGCGCTGAAGCGCGGAA GC- 3′	Ascorbic acid, dopamine, uric acid, L-lysine, L-cysteine, L-tyrosine, hypoxanthine, progesterone, and cholesterol	Serum	(Liu et al., 2019) [55]
Screen-printed carbon electrode/Carbon nanodot	NH_2-5′-GCTTCCAGCTTATTGAATTACACGCAGAGGGTA GCGGCTCTGCGCATTCAATTGCT GCGCGCTGAAGCGCG GAAGC-3'	Estriol, progesterone, and bisphenol A	River water	(Mat Zaid et al., 2020) [40]
GCE and laser-scribed graphene /poly(β-CD)/AF1-ADA/ON1/AF2-Au	The sequence of 5′-amine modified AF 1 was 5′-NH_2-AAG GGA TGC CGT TTG GG-3′; the 3′ - thiol modified AF2 sequence was 5′-CCC AAG TTC GGC ATA GTG-SH-3′; the ON 1 was 5′-AAG CTT GGG CCA TGC CCA GGA AGG ACC CAA ACG G-3′ and ON 2 :5′-CCG TTT GGG TCC TTC CTG GGC ATG GCC CAA GCT T-3′.	Glucose, bisphenol A, progesterone, and bovine serum albumin	Milk	(Chang et al., 2021) [43]

Electrode	Estradiol Specific Aptamer Sequence	Selectivity in a Matrix (Interference Effects from Various Materials)	Environmental Sample Measurements	References
SPCE/poly(pyrrole-co-pyrrole-3-carboxylic acid)	NH_2-5′-ATA CGA GCT TGT TCA ATA CGA AGG GAT GCC GTT TGG GCC CAA GTT CGG CAT AGT GTG GTG ATA GTA AGA GCA ATC-3′	N/A	N/A	(Rozi et al., 2021) [53]
PEDOT-GO/Au@Pt/Apt/MCH/GCE	5-SH-$(CH_2)_6$-GCT-TCC-AGC-TTA-TTG-AAT-TAC-ACG-CAG-AGG-GTA-GCG-GCT-CTG-CGC-ATT-CAA-TTG-CTG-CGC-GCT-GAA-GCG-CGG-AAG-C-3′	Bisphenol A, estrone, estriol, and ethinylestradiol	Serum	(Zhao et al., 2022) [76]

References

13. Rather, J.A.; Khudaish, E.A.; Kannan, P. Graphene-Amplified Femtosensitive Aptasensing of Estradiol, an Endocrine Disruptor. *Analyst* **2018**, *143*, 1835–1845. https://doi.org/10.1039/C7AN02092A.

35. Kim, Y.S.; Jung, H.S.; Matsuura, T.; Lee, H.Y.; Kawai, T.; Gu, M.B. Electrochemical Detection of 17β-Estradiol Using DNA Aptamer Immobilized Gold Electrode Chip. *Biosens. Bioelectron*. **2007**, *22*, 2525–2531. https://doi.org/10.1016/j.bios.2006.10.004.

37. Ke, H.; Liu, M.; Zhuang, L.; Li, Z.; Fan, L.; Zhao, G. A Femtomolar Level 17β-Estradiol Electrochemical Aptasensor Constructed On Hierachical Dendritic Gold Modified Boron-Doped Diamond Electrode. *Electrochim. Acta* **2014**, *137*, 146–153. https://doi.org/10.1016/j.electacta.2014.06.014.

38. Lin, Z.; Chen, L.; Zhang, G.; Liu, Q.; Qiu, B.; Cai, Z.; Chen, G. Label-Free Aptamer-Based Electrochemical Impedance Biosensor for 17β-Estradiol. *Analyst* **2012**, *137*, 819–822. https://doi.org/10.1039/C1AN15856B.

39. Zhu, B.; Alsager, O.A.; Kumar, S.; Hodgkiss, J.M.; Travas-Sejdic, J. Label-Free Electrochemical Aptasensor for Femtomolar Detection of 17β-Estradiol. *Biosens. Bioelectron*. **2015**, *70*, 398–403. https://doi.org/10.1016/j.bios.2015.03.050.

40. Mat Zaid, M.H.; Abdullah, J.; Rozi, N.; Mohamad Rozlan, A.A.; Abu Hanifah, S. A Sensitive Impedimetric Aptasensor Based on Carbon Nanodots Modified Electrode for Detection of 17ß-Estradiol. *Nanomaterials* **2020**, *10*, 1346. https://doi.org/10.3390/nano10071346.

42. Huang, K.-J.; Liu, Y.-J.; Zhang, J.-Z.; Cao, J.-T.; Liu, Y.-M. Aptamer/Au Nanoparticles/Cobalt Sulfide Nanosheets Biosensor for 17β-Estradiol Detection Using a Guanine-Rich Complementary DNA Sequence for Signal Amplification. *Biosens. Bioelectron.* **2015**, *67*, 184–191. https://doi.org/10.1016/j.bios.2014.08.010.

43. Chang, Z.; Zhu, B.; Liu, J.; Zhu, X.; Xu, M.; Travas-Sejdic, J. Electrochemical Aptasensor for 17β-Estradiol Using Disposable Laser Scribed Graphene Electrodes. *Biosens. Bioelectron*. **2021**, *185*, 113247. https://doi.org/10.1016/j.bios.2021.113247.

47. Olowu, R.A.; Arotiba, O.; Mailu, S.N.; Waryo, T.T.; Baker, P.; Iwuoha, E. Electrochemical Aptasensor for Endocrine Disrupting 17β-Estradiol Based on a Poly(3,4-Ethylenedioxylthiopene)-Gold Nanocomposite Platform. *Sensors* **2010**, *10*, 9872–9890. https://doi.org/10.3390/s101109872.

48. Fan, L.; Zhao, G.; Shi, H.; Liu, M. A Simple and Label-Free Aptasensor Based on Nickel Hexacyanoferrate Nanoparticles as Signal Probe for Highly Sensitive Detection of 17β-Estradiol. *Biosens. Bioelectron.* **2015**, *68*, 303–309. https://doi.org/10.1016/j.bios.2015.01.015.
49. Nameghi, M.A.; Danesh, N.M.; Ramezani, M.; Alibolandi, M.; Abnous, K.; Taghdisi, S.M. An Ultrasensitive Electrochemical Sensor for 17β-Estradiol Using Split Aptamers. *Anal. Chim. Acta* **2019**, *1065*, 107–112. https://doi.org/10.1016/j.aca.2019.02.062.
50. Huang, K.-J.; Liu, Y.-J.; Shi, G.-W.; Yang, X.-R.; Liu, Y.-M. Label-Free Aptamer Sensor for 17β-Estradiol Based on Vanadium Disulfide Nanoflowers and Au Nanoparticles. *Sens. Actuators B Chem.* **2014**, *201*, 579–585. https://doi.org/10.1016/j.snb.2014.05.055.
51. Huang, K.-J.; Liu, Y.-J.; Zhang, J.-Z.; Liu, Y.-M. A Novel Aptamer Sensor Based on Layered Tungsten Disulfide Nanosheets and Au Nanoparticles Amplification for 17β-Estradiol Detection. *Anal. Methods* **2014**, *6*, 8011–8017. https://doi.org/10.1039/C4AY01478B.
52. Huang, K.-J.; Liu, Y.-J.; Zhang, J.-Z. Aptamer-Based Electrochemical Assay of 17β-Estradiol Using a Glassy Carbon Electrode Modified with Copper Sulfide Nanosheets and Gold Nanoparticles, and Applying Enzyme-Based Signal Amplification. *Microchim. Acta* **2015**, *182*, 409–417. https://doi.org/10.1007/s00604-014-1352-0.
53. Rozi, N.; Hanifah, S.A.; Zaid, M.H.M.; Abd Karim, N.H.; Ikeda, M. Feasible Study on Poly(Pyrrole-Co-Pyrrole-3-Carboxylic Acid)-Modified Electrode for Detection of 17β-Estradiol. *Chem. Pap.* **2021**, *75*, 3493–3503. https://doi.org/10.1007/s11696-021-01597-9.
54. Ming, T.; Wang, Y.; Luo, J.; Liu, J.; Sun, S.; Xing, Y.; Xiao, G.; Jin, H.; Cai, X. Folding Paper-Based Aptasensor Platform Coated with Novel Nanoassemblies for Instant and Highly Sensitive Detection of 17β-Estradiol. *ACS Sens.* **2019**, *4*, 3186–3194. https://doi.org/10.1021/acssensors.9b01633.
55. Liu, X.; Deng, K.; Wang, H.; Li, C.; Zhang, S.; Huang, H. Aptamer Based Ratiometric Electrochemical Sensing of 17β-Estradiol Using an Electrode Modified with Gold Nanoparticles, Thionine, and Multiwalled Carbon Nanotubes. *Microchim. Acta* **2019**, *186*, 347. https://doi.org/10.1007/s00604-019-3465-y.
76. Zhao, Z.; Chen, H.; Cheng, Y.; Huang, Z.; Wei, X.; Feng, J.; Cheng, J.; Mugo, S.M.; Jaffrezic-Renault, N.; Guo, Z. Electrochemical Aptasensor Based on Electrodeposited Poly(3,4-Ethylenedioxythiophene)-Graphene Oxide Coupled with Au@Pt Nanocrystals for the Detection of 17β-Estradiol. *Microchim. Acta* **2022**, *189*, 178. https://doi.org/10.1007/s00604-022-05274-w.
